# Testing the role of SOX15 in human primordial germ cell fate

**DOI:** 10.12688/wellcomeopenres.15381.2

**Published:** 2019-09-23

**Authors:** Merrick Pierson Smela, Anastasiya Sybirna, Frederick C.K. Wong, M. Azim Surani

**Affiliations:** 1 Wellcome Trust/CRUK Gurdon Institute, Cambridge, CB2 1QN, UK

**Keywords:** SOX15, SOX17, PRDM14, germline, primordial germ cells, auxin-inducible degron

## Abstract

**Background: **Potentially novel regulators of early human germline development have been identified recently, including SOX15 and SOX17, both of which show specific expression in human primordial germ cells. SOX17 is now known to be a critical specifier of human germ cell identity. There have been suggestions, as yet without evidence, that SOX15 might also play a prominent role. The early human germline is inaccessible for direct study, but an *in vitro* model of human primordial germ cell-like cell (hPGCLC) specification from human embryonic stem cells (hESCs) has been developed. This enables mechanistic study of human germ cell specification using genetic tools to manipulate the levels of SOX15 and SOX17 proteins to explore their roles in hPGCLC specification.

**Methods: **SOX15 and SOX17 proteins were depleted during hPGCLC specification from hESCs using the auxin-inducible degron system, combined with a fluorescent reporter for tracking protein levels. Additionally, SOX15 protein was overexpressed using the ProteoTuner system. Protein-level expression changes were confirmed by immunofluorescence. The impact on hPGCLC specification efficiency was determined by flow cytometry at various time points. qPCR experiments were performed to determine some transcriptional effects of SOX15 perturbations.

**Results: **We observed specific SOX15 expression in hPGCLCs by using immunofluorescence and flow cytometry analysis. Depletion of SOX15 had no significant effect on hPGCLC specification efficiency on day 4 after induction, but there was a significant and progressive decrease in hPGCLCs on days 6 and 8. By contrast, depletion of SOX17 completely abrogated hPGCLC specification. Furthermore, SOX15 overexpression resulted in a significant increase in hPGCLC fraction on day 8. qPCR analysis revealed a possible role for the germ cell and pluripotency regulator PRDM14 in compensating for changes to SOX15 protein levels.

**Conclusions: **SOX17 is essential for hPGCLC specification, yet SOX15 is dispensable. However, SOX15 may have a role in maintaining germ cell identity.

## Introduction

Despite decades of research, the genetic regulation of mammalian germline development is still only partially understood, especially in the case of the human germline. Primordial germ cells (PGCs) are the founder cells of sperm and eggs, which are specified shortly after blastocyst-implantation and preceding gastrulation ^1^. This stage of human development is inaccessible for direct study, although experiments in mice and other animals have identified some important regulatory factors ^2^. Later stages of human PGC (hPGC) development, when they migrate to the developing gonad, have been studied using fetal tissue samples, but these samples are highly limited in their availability and cannot be genetically manipulated.

More recently, a model system has been developed in which pluripotent stem cells can be induced to differentiate into PGC-like cells (PGCLCs) in response to signaling by BMP and other cytokines ^3^. Mouse PGCLCs can develop further *in vitro* when co-cultured with E12.5 ovarian somatic cells in the presence of a defined set of cytokines and hormones; a few of them can even develop into functional oocytes ^4^. Although a similar system has been recently reported to produce small numbers of human oogonia using coculture with mouse fetal ovarian somatic tissue ^5^, the precise factors required for hPGC maturation and epigenetic resetting remain unknown, and the current human PGCLC (hPGCLC) model system only allows study of cells in the pre-migratory state ^3, 6^.

Solving this problem requires understanding the differences in gene regulation between hPGCs and mPGCs. In mice, PGC fate is specified by a core network of three transcription factors: BLIMP1, PRDM14, and AP2γ ^2, 7^. These genes are also important in hPGCs ^8, 9^, but their activities depend on SOX17, which is the crucial specifier of hPGC fate ^6^. Indeed, pigs, which are not closely related to primates, rely on SOX17 for germline specification ^10^, suggesting that the SOX17-driven mode of PGC specification is likely to represent a pathway conserved among non-rodent mammals ^11^.

SOX17 is not the only regulatory gene that differs between mPGCs and hPGCs. SOX15, another member of the SOX family, is strongly expressed in hPGCs, but is absent in mPGCs ^12^. Indeed, a recent single-cell transcriptomics study on human fetal PGCs found that expression of *SOX15* mRNA was stronger and relatively more homogenous than *SOX17* among hPGCs before 10 weeks’ gestation, and the authors claimed that SOX15 is probably functionally more important for hPGC development *in vivo* ^13^.

In both mice and humans, SOX15 is highly expressed in naïve ESCs, placenta, and muscle satellite cells ^14^. Loss of SOX15 function in mice produces a relatively mild phenotype, except for impaired muscle regeneration after injury ^15^. Notably, fertility is normal, ruling out a crucial role for SOX15 in mPGCs. The SOX factors are classified into groups A–H based on phylogenetic analysis of their high-mobility group (HMG) DNA-binding domains ^16^. SOX15 is the only group G SOX factor in mammals, although its HMG domain is similar to that of group B SOX factors such as SOX2 ^16^. Interestingly, SOX2 is expressed in mPGCs but not hPGCs ^17, 18^, whereas SOX15 shows the opposite pattern ^12, 13^. In mESCs, SOX2 knockout, which causes differentiation and loss of pluripotency, can be rescued by overexpression of SOX15 ^19^. In contrast, SOX17 overexpression causes differentiation to endodermal lineages, even when SOX2 is present as normal ^20^. Furthermore, in both mice and humans the structures of SOX15 and SOX17 are relatively dissimilar ^16^. Although SOX15 and SOX17 have similar expression patterns in hPGCs ^13^, their transcriptional roles may differ.

In this work, we investigated the role of SOX15 during the specification of hPGCLCs *in vitro* and compared it with SOX17. To do this, we manipulated levels of these proteins using the auxin-inducible degron (AID) ^21^ and ProteoTuner ^22^ systems, which allow for tight protein-level control with good temporal resolution. The AID system involves fusing a short degron peptide to the protein of interest, and also expressing a TIR1 E3 ubiquitin ligase ^21, 23^. In the presence of auxin (indole-3-acetic acid (IAA)), TIR1 will ubiquitylate the degron, leading to destruction of the target protein by the proteasome. This happens rapidly, causing complete depletion within one hour. The presence of a Venus fluorescent reporter tag is compatible with AID, and this combination has previously been used successfully in hPGCLCs ^8^. In accordance with the known role for SOX17 in the human germline, we found that its depletion prevented hPGCLC specification. In contrast, we found that SOX15 is dispensable for establishing hPGCLC identity, but may play a role in maintaining it. Furthermore, we identified some transcriptional effects of SOX15 depletion and overexpression. Altogether, we show the utility of genetic tools, which rapidly alter protein levels, for providing insights into genetic regulation of the early human germline.

## Methods

### Cell culture

hESCs (WIS2 (46XY) cell line ^24^ obtained from the Weizmann Institute, with NANOS3-T2A-tdTomato (N3tdT) reporter subsequently introduced ^10^) were cultured in 4i medium ^6, 24^, containing cytokines TGFβ, bFGF, and LIF, as well as four small-molecule inhibitors for kinases MAPK, MEK, JNK, and GSK3. This medium, which was prepared as previously described ^6^, allows hESCs to be continually maintained in a germline competent state. The hESCs were grown on a layer of irradiated CF1 mouse embryonic fibroblasts (MEFs) (Applied Stem Cell). The MEFs were plated at approximately 15,000 cells/cm ^2^ on gelatin-coated plates in DMEM supplemented with 10% FBS, 100 U/mL penicillin, and 0.1 mg/mL streptomycin. Medium was changed daily for hESCs. Passages were performed using 0.25% trypsin/EDTA with ROCK inhibitor (10 µM Y-27632, Tocris Bioscience) added to the medium. All cells were maintained in an incubator at 37°C and 5% CO _2_. Cell lines used in the experiments tested negative for mycoplasma.

### Generation of mutant cell lines

For CRISPR/Cas9 experiments, gRNAs were chosen using the online tool at crispr.mit.edu (accessed October 2018; this resource is no longer functional but contains links to multiple free-to-use alternatives). Oligos were annealed and cloned into eSpCas9(1.1) vector ^25^ digested with BbsI. Homology arms (approximately 1 kb each) were amplified by PCR from genomic DNA of the target cell line, with a point mutation introduced to remove the stop codon and CRISPR PAM. The homology-directed repair donor plasmids were assembled using InFusion cloning (Clontech). For ProteoTuner overexpression, SOX15 cDNA was cloned into the PB-EF1-myc-DD-IRES-Puro backbone using InFusion. This plasmid was stably integrated into N3tdT hESCs using the PiggyBac system ^8^. Full plasmid sequences with annotation are listed in the *Extended data* ^26^. All oligos used for cloning and sequencing are listed in Extended Table 1 ^27^. Plasmids were delivered using Lipofectamine Stem reagent (Invitrogen) according to manufacturer’s instructions. After 48 hours, selection was begun with puromycin (0.5 µg/mL), hygromycin (50 µg/mL), and/or FIAU (200 nM) as appropriate, and continued until colonies were picked. Genotyping gels for AID knock-ins are shown in Extended Figure 1 ^28^. After the AID tag was introduced, cells were subsequently transfected with TIR1 using the PiggyBac system ^8^, and the selectable marker was excised using transient expression of Dre recombinase ^29^.

### hPGCLC induction

For hPGCLC induction, hESCs cultured in 4i medium were dissociated with 0.25% trypsin/EDTA. The cells were suspended in MEF medium to quench the trypsin, and the suspension was filtered through a 50-µm strainer. The cells were pelleted by centrifugation (300 *g*, 4 minutes) and resuspended in hPGCLC base medium ^10^ (Advanced RPMI 1640 (Thermo Fisher), supplemented with 2 mM L-glutamine, 0.1 mM non-essential amino acids, 1% B27 supplement (Thermo Fisher), 100 U/mL penicillin, 0.1 mg/mL streptomycin, 10 µM Y-27632, 0.25% w/v poly(vinyl alcohol), and 10 ng/mL hLIF). Cells were counted (Invitrogen Countess) and the suspension was diluted to 40,000 live cells/mL in hPGCLC medium (hPGCLC base plus 500 ng/mL BMP2, 100 ng/mL SCF, and 50 ng/mL EGF). Next, 100 µL of suspension, containing 4000 cells, was added to each well of a 96-well ultra-low-attachment plate (Corning CoStar). Cells were pelleted (300 *g*, 2 minutes) and the plate was incubated (37°C, 5% CO _2_). For experiments beyond day 6 of culture, a 50% medium change was performed on day 6. Details of each separate biological replicate (144 in total), including timepoints, are listed in Extended Table 3 (for SOX15 and SOX17 AID experiments), Extended Table 4 (for SOX15-AID time course experiments), or Extended Table 5 (for SOX15-DD experiments) ^27^.

### AID experiments

For AID, IAA sodium salt was prepared as a stock solution in water (500 mM) and added to the cell culture medium at a final concentration of 100 µM. In SOX15-AID time-course experiments where the IAA was added after induction, 10 µL of 1.1 mM IAA in hPGCLC base medium were added. In these experiments, 10 µL of hPGCLC base medium containing no IAA were also added to control wells. For ProteoTuner experiments, Shield1 was used at a concentration of 0.5 µM.

### Flow cytometry

Embryoid bodies (EBs) were collected, washed with PBS, and dissociated by digesting with 0.25% trypsin/EDTA (5 µL per EB) for 10 minutes at 37°C with gentle shaking (600 rpm). For day 6 and older EBs, dissociation was completed by passing the suspension multiple times through a 27-gauge needle. Trypsin was quenched with two volumes of ice-cold sorting medium (3% FBS in PBS) and the cells were pelleted (300 *g*, 2 minutes). Next, the cells were resuspended in sorting medium (5 µL per EB) containing a 1:60 dilution of AF647 conjugated mouse anti-human tissue non-specific alkaline phosphatase IgG (BD Biosciences, catalog No. 561500, RRID AB_10717125) and incubated at 4°C in the dark for 30 minutes. The antibody solution was diluted with two volumes of sorting medium, and the cells were pelleted (300 *g*, 2 minutes) and resuspended in 500 µL sorting medium plus DAPI (0.1 µg/mL). The suspension was filtered with a 50 µm strainer and analyzed on a flow cytometer (BD LSRFortessa or Sony 800Z). Cells in FACS experiments were sorted directly into 50 µL RNA extraction buffer (Arcturus PicoPure, Thermo Fisher) which was frozen at –80°C for subsequent use. RNA was extracted following the manufacturer’s instructions.

### Quantitative reverse-transcription PCR (qPCR)

cDNA synthesis was performed using the Quantitect Reverse Transcription kit (Qiagen) following the manufacturer’s instructions. qPCR reactions were performed at 10 µL scale in 384-well plate format using the SYBR Green JumpStart Taq ReadyMix (Sigma-Aldrich) on a QuantStudio 6 Flex instrument (Applied Biosystems). The following protocol was used for thermocycling: initial denaturation 95°C, 10 minutes, followed by 40 cycles of 95°C denaturation for 15 seconds and 60°C annealing/extension for 1 minute. Primers are listed in Extended Table 1 ^27^. Two technical replicates were performed for each biological replicate. Details, including mean Ct values, for all biological replicates (80 in total) are listed in Extended Table 9 ^27^. Analysis was performed using the QuantStudio software. The ∆∆Ct method was used for quantification, with *GAPDH* as a reference transcript.

### Immunofluorescence

For immunofluorescence in hESCs, cells were grown on Ibidi 8-well plates. Cells were washed with PBS, fixed with 4% paraformaldehyde in PBS at room temperature (RT) for 10 minutes, and washed again with PBST (0.1% Triton X-100 (Sigma) in PBS) three times. Cells were permeabilized with 0.25% Triton X-100 (Sigma) in PBS for 10 minutes, then blocked with blocking buffer (5% normal donkey serum (Stratech) and 1% bovine serum albumin (Sigma) in PBST) at RT for 30 minutes. Cells were incubated with primary antibodies (Extended Table 2) ^27^ in blocking buffer overnight at 4°C, then washed three times with PBST. Cells were incubated with secondary antibodies (Extended Table 2) ^27^ in blocking buffer at RT for one hour, then incubated with 0.5 ng/mL DAPI in PBS for 10 minutes. Cells were washed three times with PBS and stored at 4°C in the dark until imaging (up to one week).

For immunofluorescence in EBs, the EBs were fixed with 4% paraformaldehyde in PBS at 4°C for 1 hour, then washed with PBS and transferred to 10% sucrose in PBS. When EBs had sunk, the process was repeated with 20% sucrose. EBs were then embedded in OCT compound (CellPath) and cryosectioned using a Leica CM3050S cryostat to 8-µm thickness on SuperFrost Plus slides (VWR). Slides were air-dried for 1 hour at RT, then stained for immunofluorescence as described above, except that the permeabilization was performed with 0.1% Triton X-100 and the DAPI was added during secondary antibody incubation. Slides were mounted in Prolong Gold Antifade medium with DAPI (Molecular Probes). Imaging for all samples was performed with an SP5 confocal laser scanning microscope (Leica) and images were analyzed using Fiji software (version 2.0.0) ^30^.

### Statistical analysis

For flow cytometry data, the hPGCLC fraction (listed in Extended Tables 3–5) ^27^ was calculated using FlowJo software (version 10.0.7). For each induced cell line, the fold change was calculated as the ratio of hPGCLC fraction in treated (with IAA or Shield1, depending on experiment) and untreated samples. This step was performed in order to control for the batch-to-batch variability between different hPGCLC inductions. The fold change values were then compared for experimental cell lines (overexpression or depletion) and control cell lines (either SOX15-AID-Venus with no TIR1, or parental N3tdT). This was done in order to control for any nonspecific effects of Shield1 or IAA (a known aryl hydrocarbon receptor agonist ^31^). The Wilcoxon rank-sum test was used for comparisons, since by the Kolmogorov-Smirnov test the data were not normally distributed.

For qPCR data, differentially expressed genes were determined by Z-test on the ∆∆Ct data. This test was chosen because the data did not significantly deviate from normality by the Kolmogorov-Smirnov test. The Holm-Bonferroni method was used to correct for multiple comparisons. All data points were included in analysis, except those from reactions that did not amplify due to low target concentration.

Statistical analysis was performed in R (version 3.2.3) using the RStudio environment (version 1.2.1335). p < 0.05 was used as a significance threshold for all tests. Sample sizes were not determined in advance. Investigators were not blinded during experiments and analysis.

## Results

### Depletion of SOX15 during hPGCLC specification using AID

**Figure 1.**
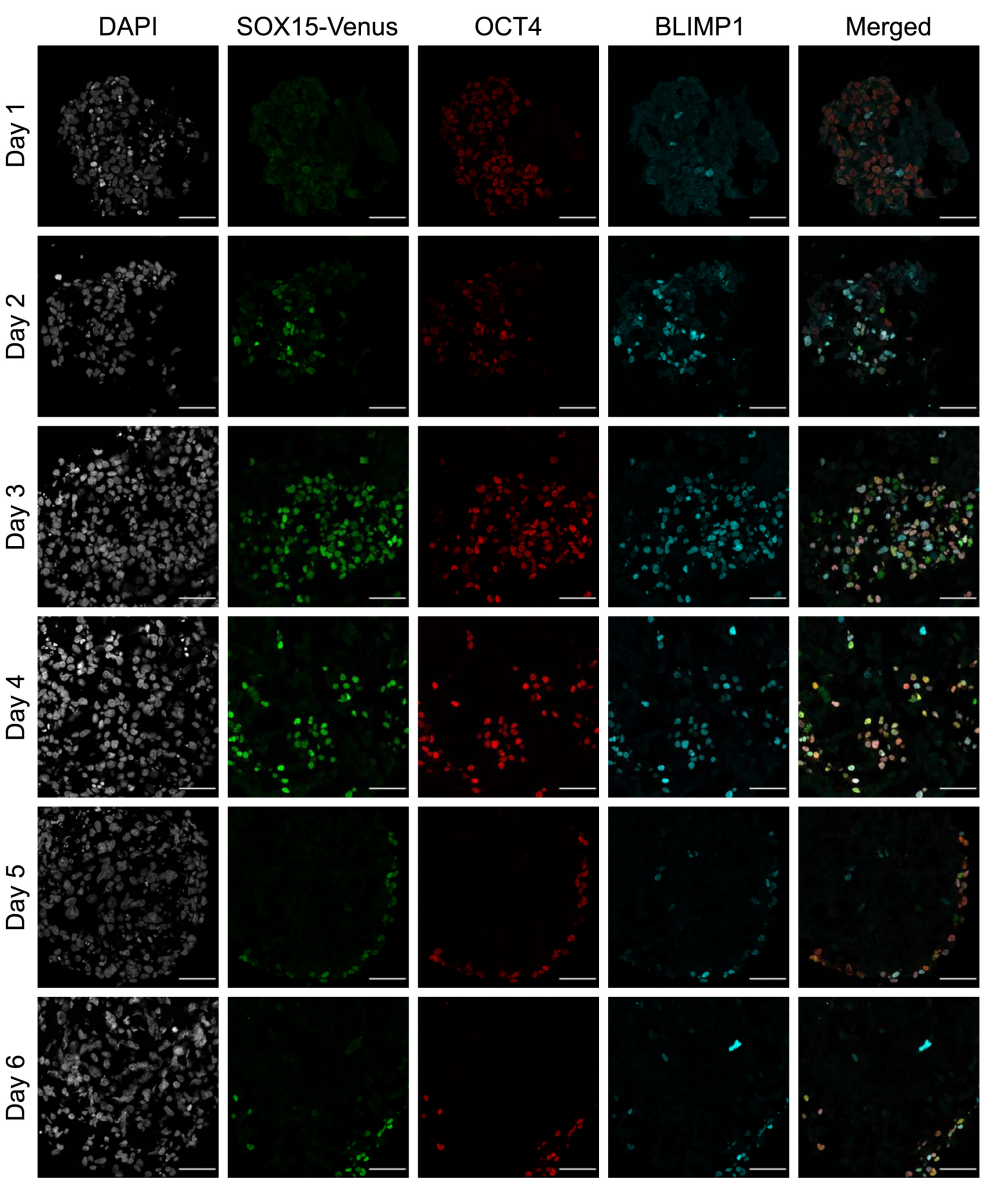
Timecourse immunofluorescence of SOX15-AID-Venus embryoid bodies (EBs). EBs were stained with DAPI (grey), anti-Venus (green), anti-OCT4 (red), and anti-BLIMP1 (cyan). Scale bar is 50 µm. SOX15 expression is observed faintly on day 1 and robustly on day 2, persisting in OCT4/BLIMP1 positive cells for the remainder of the experiment.

To determine the effects of SOX15 depletion on hPGCLC specification, a homozygous knock-in cell line with a C-terminal AID-Venus tag on SOX15 was generated by a strategy similar to one previously used for PRDM14 ^8^. The parental line had a NANOS3-T2A-tdTomato (N3tdT) reporter, which is expressed specifically in hPGCLCs ^10^. Immunofluorescence experiments confirmed SOX15 expression at the protein level in hPGCLCs within the EBs ( Figure 1); this is consistent with the previous RNA-seq data ^6, 13^. The expression of SOX15 was first observed at a faint level on day 1 after induction, and more strongly on day 2. Expression continued in OCT4/BLIMP1-positive hPGCLCs until the end of the time-course experiment (day 6) ( Figure 1). The neighboring somatic lineages (soma) were almost completely SOX15-negative: there were a small number of SOX15-positive, OCT4/BLIMP1 negative cells on days 2–4, but by day 5, SOX15 expression was completely confined to hPGCLCs.

**Figure 2.**
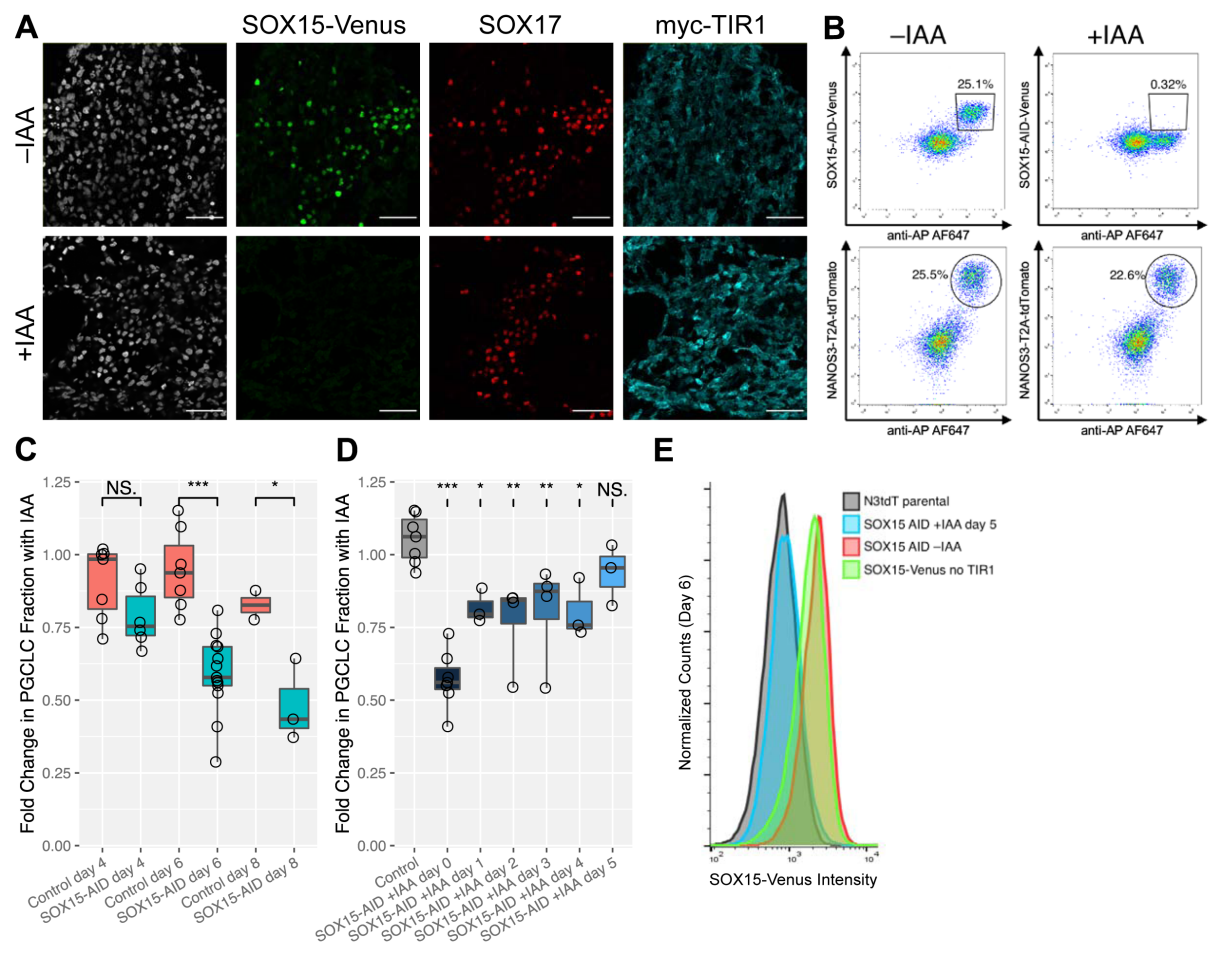
SOX15 depletion by auxin-inducible degron (AID). ( **A**) SOX15-AID-Venus expression is observed in SOX17-positive human primordial germ cell-like cells (hPGCLCs). A few SOX17-positive Venus-negative somatic cells are also present; these are likely definitive endoderm ^6^. SOX15-AID-Venus, but not SOX17, is depleted to background levels with indole-3-acetic acid (IAA) treatment. Staining for TIR1-myc indicates ubiquitous expression. Scale bar is 50 µm. ( **B**) Representative flow cytometry analysis of SOX15-AID-Venus protein expression and hPGCLC markers. hPGCLCs were identified based on NANOS3-T2A-tdTomato expression and AP surface staining. IAA treatment results in a near-total reduction of Venus-positive cells on day 4. However, this only causes a slight decrease in the fraction of AP+/NANOS3+ hPGCLCs. ( **C**) Prolonged SOX15 depletion decreases AP+/NANOS3+ hPGCLC fraction. Embryoid bodies were treated with IAA from the start of induction, with hPGCLC fraction measured by flow cytometry on day 4, 6, or 8. Fractions were normalized with respect to untreated samples of the same clones. Statistical comparisons were performed between SOX15-AID/TIR1 clones and control clones without TIR1, which did not deplete SOX15. ( **D**) Effects of SOX15 depletion at various times during hPGCLC specification. IAA treatment was begun on the day indicated, with AP+/NANOS3+ hPGCLC fraction measured on day 6. Fractions were normalized with respect to untreated samples of the same clones. Comparisons shown are relative to control cell lines (without TIR1). Significance values are by Wilcoxon test (*p < 0.05, **p < 0.01, ***p < 0.001). Each point represents a biological replicate. ( **E**) Venus fluorescence intensity in NANOS3+/AP+ PGCLCs was measured by flow cytometry. In the absence of IAA, SOX15-AID-Venus fluorescence was similar to its levels in the parental cell line without TIR1. This shows that TIR1 does not cause leaky depletion of SOX15. When IAA was added on day 5, by day 6 the Venus fluorescence was depleted to background levels, similar to the N3tdT cell line which lacks Venus completely.

To deplete SOX15, SOX15-AID/TIR1 cells were treated with IAA at the start of hPGCLC induction. Immunofluorescence performed on day 4 after induction showed depletion of SOX15-AID-Venus to background levels ( Figure 2A). SOX15-AID-Venus expression in untreated cells was similar to that observed in the previous experiment. SOX17 was used as a marker for hPGCLCs, and SOX17-positive cells were present in both samples. To quantify any effects of SOX15 depletion on hPGCLC specification efficiency, flow cytometry was performed on cells from dissociated EBs either treated or untreated with IAA. Identification of hPGCLCs was performed using a combination of the NANOS3-T2A-tdTomato reporter and antibody staining against the alkaline phosphatase surface marker. The results indicated that on day 4 after induction, there was no significant effect of SOX15 depletion on induction efficiency ( Figure 2B). However, at later time points (days 6 and 8), depletion of SOX15 resulted in a significant reduction of the fraction of hPGCLCs ( Figure 2C). Notably, the effect was much milder that that reported for SOX17 depletion, which resulted in complete loss of hPGCLCs ^6, 8^. The progressive decrease in hPGCLC fraction upon prolonged SOX15 depletion suggests that SOX15 may have a role in hPGCLC maintenance.

The rapid kinetics of SOX15 depletion by the AID system enabled an investigation of the effects of SOX15 depletion starting at various time points after induction. IAA was added on days 0 through 5 and the hPGCLC fraction present on day 6 ( Figure 2D) was measured by flow cytometry, which also confirmed SOX15 depletion even after only one day of IAA treatment ( Figure 2E). As expected, the effect on hPGCLC fraction diminished when IAA was added at later time points, but there was a significant decrease on day 6 when SOX15 was depleted starting on day 4 or earlier. There were no statistically significant differences in the magnitude of these decreases with SOX15 depleted from day 4 or earlier (Wilcoxon test, p > 0.05). Interestingly, depletion from day 0 did not produce a significant effect on hPGCLC fraction measured on day 4 ( Figure 2C), but depletion from day 4 significantly reduced hPGCLC fraction measured on day 6 (Wilcoxon test, p = 0.02). Since the hPGCLC transcriptional network is already largely established by day 2 ^6^, this suggests that SOX15 depletion might interfere with hPGCLC survival or proliferation even when specification proceeds normally.

### SOX15 overexpression experiments using ProteoTuner

To further elucidate the functional role of SOX15 in hPGCLC specification, we performed overexpression of SOX15 using the ProteoTuner system ^22^. This consists of a destabilizing domain (DD) fused to the protein target, which normally results in protein degradation. Upon addition of a stabilizing ligand (Shield1), protein levels increase quickly. The ProteoTuner system has rapid kinetics ^22^ similar to those of the AID system ^21^.

**Figure 3.**
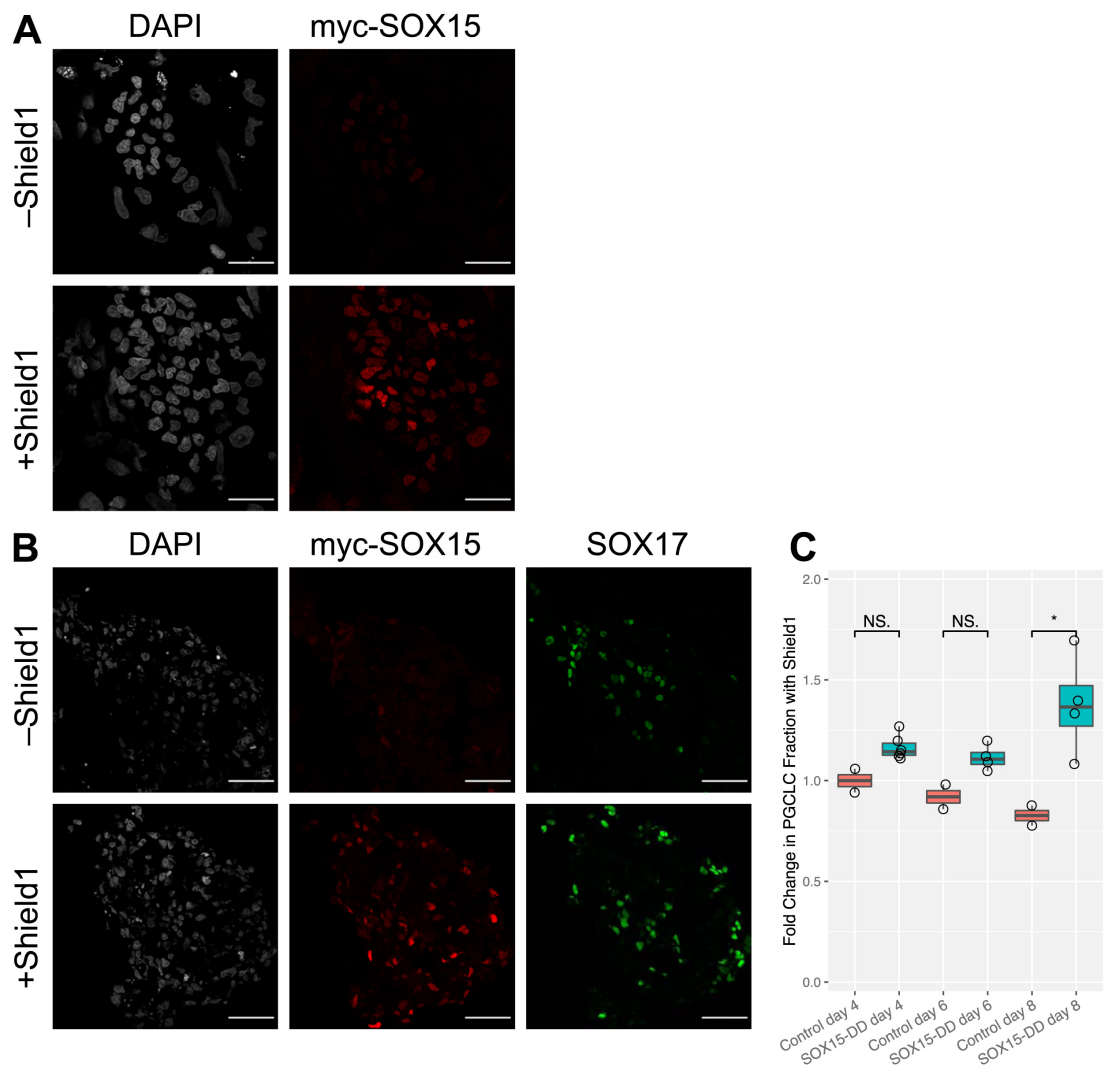
SOX15 overexpression by ProteoTuner. ( **A**) Human embryonic stem cells (hESCs) express myc-SOX15-DD after one hour of treatment with Shield1. hESC colonies were grown on a layer of MEFs and stained for IF using anti-myc antibody. myc-SOX15-DD successfully localizes to the nucleus. Scale bar is 50 µm. ( **B**) In a Shield1-dependent manner, EBs overexpress myc-SOX15-DD in both SOX17-positive human primordial germ cell-like cells (hPGCLCs) as well as somatic cells. Scale bar is 50 µm. ( **C**) SOX15 overexpression increases hPGCLC fraction. EBs were treated with Shield1 starting at the beginning of induction, and AP+/NANOS3+ hPGCLC fraction was measured by flow cytometry on day 4, 6, or 8. Fractions were normalized with respect to untreated samples of the same clones. Significance values are by Wilcoxon test (*p < 0.05, **p < 0.01, ***p < 0.001). Each point represents a biological replicate.

hESC cell lines were generated expressing SOX15 with *N-*terminal myc tag and *C-*terminal DD, under the control of the constitutively active EF1α promoter. After selection, clones were tested for Shield1-dependent expression by immunofluorescence after 1 hour of treatment ( Figure 3A). Two suitable clones were identified with homogeneous expression of myc-SOX15-DD protein, observed only in the presence of Shield1. Subsequently, these cells were induced to form hPGCLCs in the presence or absence of Shield1. On day 4 post-induction, Shield1 treatment resulted in myc-SOX15-DD expression in both hPGCLCs and soma as judged by immunofluorescence ( Figure 3B).

To quantify the effect of SOX15 overexpression on hPGCLC induction efficiency, the EBs were dissociated and analyzed by flow cytometry. Notably, there was a higher fraction of hPGCLCs in EBs overexpressing SOX15 ( Figure 3C). This difference was statistically significant on day 8 (Wilcoxon test, p = 0.02), which was not the case on days 4 and 6. Taken together with the delayed effects of SOX15 depletion observed in the AID experiments, this further supports a potential role for SOX15 in hPGCLC maintenance, possibly by promoting survival or proliferation.

### Transcriptional effects of SOX15 perturbation

**Figure 4.**
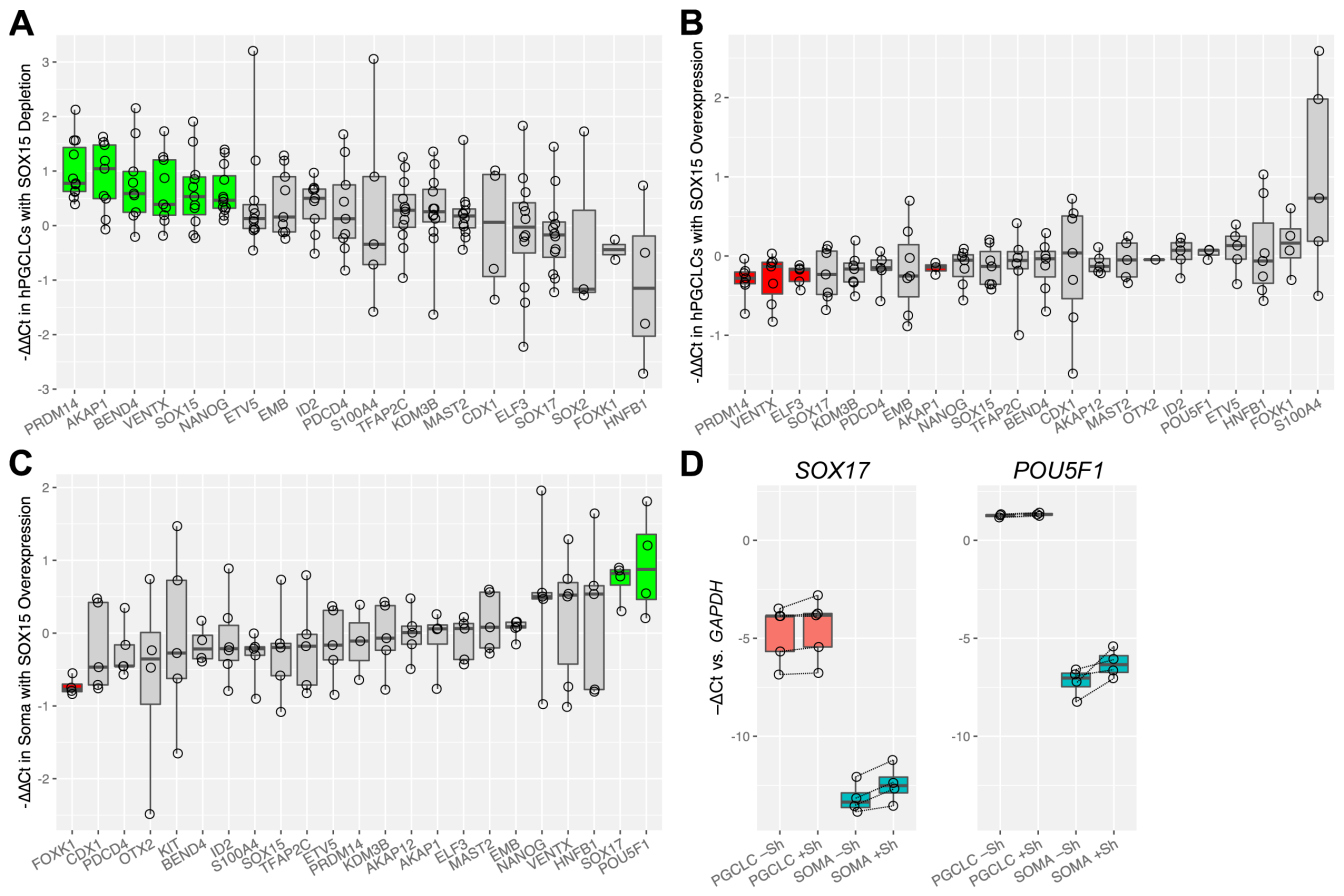
Transcriptional effects of SOX15 perturbation. Expression of candidate genes was measured by qPCR to determine the effects of ( **A**) SOX15 depletion from human primordial germ cell-like cells (hPGCLCs) ( **B**) SOX15 overexpression in hPGCLCs ( **C**) SOX15 overexpression in soma. The cell populations were separated by flow cytometry prior to RNA extraction. Each point represents a biological replicate. Green bars represent significantly upregulated genes, and red bars significantly downregulated ones (Z-test with Holm-Bonferroni correction, p < 0.05). ( **D**) Relative expression levels of *SOX17* and *POU5F1* in hPGCLCs and soma with and without SOX15 overexpression.

To test for transcriptional effects of SOX15 depletion and overexpression, we assembled a set of candidate genes including both known regulators of germline identity, and previously reported SOX15 targets in other cell types, including human embryonal carcinoma cells ^32^, muscle satellite cells ^33^, esophageal ^34^ and pancreatic ^35^ adenocarcinomas, and mouse ESCs ^36^. We investigated transcriptional changes for these genes in hPGCLCs after SOX15 protein was either depleted or overexpressed. We used the day 6 timepoint for experiments based on our hPGCLC analysis described above. Depletion of SOX15 during hPGCLC specification caused significant upregulation of *PRDM14, AKAP1, BEND4, VENTX, SOX15*, and *NANOG* as shown by qPCR analysis ( Figure 4A) (Z test with Holm-Bonferroni correction, p < 0.05); *PRDM14, VENTX, SOX15*, and *NANOG* are known to be associated with germ cell identity ^1, 2^, perhaps suggesting a compensatory effect. Notably, the upregulation of *SOX15* implies negative feedback, while *AKAP1, VENTX,* and *BEND4* are known targets of PRDM14 in hPGCLCs ^8^, so their upregulation may be indirect.

In contrast, hPGCLCs with SOX15-DD overexpression showed transcriptional changes that were generally the opposite of the SOX15-AID hPGCLCs ( Figure 4B). *PRDM14, AKAP1, VENTX*, and *ELF3* were significantly downregulated (Z test with Holm-Bonferroni correction, p < 0.05). The first three of these genes were upregulated in SOX15-AID. Interestingly, SOX15-DD overexpression also had distinct effects in the somatic cells of the EBs. *FOXK1,* a transcription factor known to promote aerobic glycolysis ^37^, was significantly downregulated. Additionally, *SOX17* and *POU5F1* (encoding OCT4) were significantly upregulated, showing an approximately twofold increase ( Figure 4C), although their initial expression in untreated somatic cells was low ( Figure 4D). These genes are highly expressed in hPGCLCs, and *SOX17* in particular is crucial for establishing their identity ^6^.

### AID depletion of SOX17 using inducible TIR1

**Figure 5.**
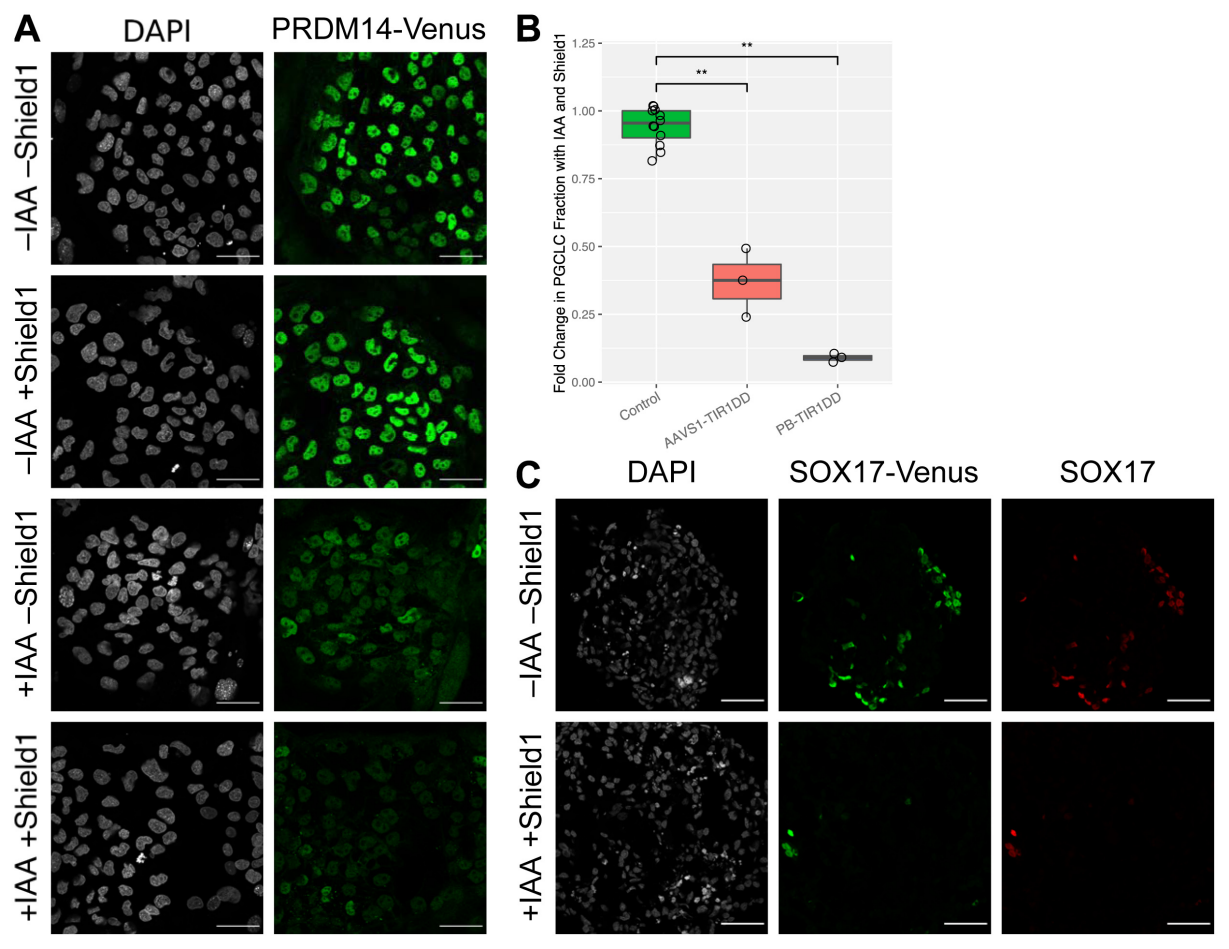
AID depletion of PRDM14 and SOX17 using inducible TIR1. ( **A**) PRDM14-AID-Venus/TIR1-DD human embryonic stem cells (hESCs) grown on a layer of mouse embryonic fibroblasts were treated with indole-3-acetic acid (IAA) and/or Shield1. After one hour of treatment, cells were fixed and stained for IF using anti-GFP. Scale bar is 50 µm. ( **B**) SOX17 depletion abrogates hPGCLC induction. SOX17-AID-Venus hESCs, expressing TIR1-DD either from AAVS1 or from random PiggyBac integration, were induced to form hPGCLCs with and without Shield1 and IAA. AP+/NANOS3+hPGCLC fraction was measured by flow cytometry after four days. Fractions were normalized with respect to untreated samples of the same clones. Results from cells without TIR1 are plotted for comparison. Significance values are by Wilcoxon test (*p < 0.05, **p < 0.01, ***p < 0.001). Each point represents a biological replicate. ( **C**) SOX17 depletion by AID with TIR1-DD. SOX17-AID-Venus cells with PB-TIR1-DD were induced to form human primordial germ cell-like cells (hPGCLCs) and treated with IAA and Shield1 from the start of the induction. After four days, EBs were fixed, sectioned, and stained using antibodies against GFP and SOX17. The untreated sample contains many SOX17 positive hPGCLCs, whereas only a few are visible in the treated sample. Scale bar is 50 µm.

SOX17 is known to play a crucial role in PGC specification and is expressed from an early stage in the process. Previous AID experiments on SOX17 have shown that its depletion abrogates hPGCLC specification ^8^. However, the cell lines used in those experiments had poor hPGCLC induction efficiency (~5%) even in the absence of IAA. We hypothesized that this was due to depletion of SOX17 even in absence of IAA. Similar leaky depletion has been previously reported for a few targets ^38^. To overcome this leakiness, we created cell lines expressing TIR1 under the control of the ProteoTuner system (TIR1-DD). In this inducible AID system, protein target depletion should occur upon administration of two ligands: Shield1 to stabilize auxin hormone receptor (TIR1) and IAA to initiate target degradation. We performed a preliminary test of kinetics in PRDM14-AID-Venus hESCs ^8^. By immunofluorescence, a TIR1-DD hESC line depleted PRDM14 after 1 hour of treatment with IAA and Shield1 ( Figure 5A), with minor heterogeneity.

We next applied the inducible TIR1-DD to SOX17-AID by generating a cell line with EF-TIR1-DD, flanked by insulator sequences to prevent silencing ^39^, knocked in to the *AAVS1* locus. This cell line successfully formed hPGCLCs with efficiency 8–17% (Extended Table 3) ^27^. Although this was not quite as efficient as wild-type SOX17 cell lines (typically 30–50%), it still was an improvement over constitutive TIR1 cell lines, which at best gave roughly 5% efficiency ^8^, and often gave less. However, the AAVS1-TIR1DD cells showed only a moderate depletion of SOX17 with IAA and Shield1 treatment ( Figure 5B). Apparently the two copies of TIR1-DD at the AAVS1 locus were insufficient, or possibly silenced despite the insulators ^40^, so we used PiggyBac transposase to deliver additional copies.

After screening clones, we identified three that were competent for hPGCLC specification but also depleted SOX17 almost completely with IAA and Shield1. As expected, this resulted in drastically reduced specification efficiency ( Figure 5B) The efficiency in the absence of IAA and Shield1 was similar to the AAVS1-TIR1DD cell line. Notably, the few remaining hPGCLCs were all SOX17-positive by flow cytometry and immunofluorescence ( Figure 5C). This indicates that the presence of these hPGCLCs was due to slightly heterogeneous depletion, rather than SOX17 being dispensable. These data further confirm the crucial role of SOX17 for hPGCLC fate.

## Discussion

Based on previous single-cell RNA-seq data, SOX15 had been suggested to be a critical regulator of human germ cell identity ^13^. In hPGCs between gestational weeks 4 and 10, SOX15 is more homogeneously expressed than SOX17 ^13^. However, AID-mediated depletion of SOX15 during hPGCLC specification did not result in significant reduction in specification efficiency, as would be expected if it were essential at this early stage. Unlike the dramatic effect seen with SOX17 depletion ( Figure 5B), SOX15 depletion only resulted in a moderate decrease in hPGCLC fraction, and this effect was only significant at later timepoints (days 6 and 8) ( Figure 2C). Furthermore, by immunofluorescence, robust expression of SOX15 is detected only after day 2 of hPGCLC induction ( Figure 1). This is later than the expression of SOX17 and BLIMP1, which are the key regulators of human germline fate ^6^, so cells are already committed to the germ lineage by this time. These results do not support a critical role for SOX15 in germline specification.

In contrast to SOX15, SOX17 is strictly required for hPGCLC specification. As expected based on previous experiments ^6, 8^, depletion of SOX17 by AID resulted in a dramatic decrease in hPGCLC fraction. While an inducible TIR1-DD was required to overcome leakiness, this system had similar kinetics to the conventional AID system ( Figure 5A), and the use of inducible TIR1-DD should not change the interpretation of the results. In addition to validating previous results with SOX17-AID ^8^, these experiments serve as a proof of concept for an inducible AID system, where TIR1 is under ProteoTuner control. Such configuration can be of great value for some targets that are destabilized by AID even in the absence of IAA, as was the case for SOX17, thus expanding the utility of the AID system.

Although not absolutely necessary for hPGCLC specification, SOX15 may, however, play a role in maintenance of germ cells. AID experiments showed that prolonged SOX15 depletion decreased the hPGCLC fraction in EBs, with the effect increasing over time ( Figure 2C). Furthermore, overexpression of SOX15 increased the hPGCLC fraction, again with the effect increasing over time ( Figure 3C). Limitations in current methods for culturing hPGCLCs make it difficult to obtain meaningful results beyond day 8 due to degeneration of the EBs, but it may well be the case that SOX15 is required for long-term maintenance of germ cell identity, similar to its role in myogenic progenitors ^15, 33, 41, 42^ in which it prevents premature differentiation. Alternatively, the effects on hPGCLC fraction could reflect a role in survival or proliferation of hPGCLCs.

We also observed transcriptional changes in response to SOX15 perturbation. The expression of *PRDM14* and some of its known target genes ^8^ were anticorrelated with SOX15 protein levels in hPGCLCs ( Figure 4). Furthermore, the effects on *SOX15* RNA levels in the AID experiments indicate negative feedback, which is possibly analogous to the feedback previously reported for *Sox2* in mESCs ^43^. The magnitude of the effects were however relatively modest (1.4 – 2.0 average fold change for the transcripts affected) within a small subset of the transcriptome we tested. We cannot therefore exclude a possibility that other SOX15 targets might be functionally relevant to a greater or lesser extent.

The effects of SOX15 perturbation on hPGCLC maintenance and transcriptional activity are better understood in context of its role in other cell types. Although SOX15 has not been investigated nearly as much as other SOX factors, the existing research on SOX15 suggests a role related to preventing improper growth and differentiation. In myogenic progenitors, SOX15 promotes satellite cell maintenance, and thus has an important role in muscle regeneration ^15, 33, 41, 42^. In embryonal carcinoma ^32^, and esophageal ^34^ and pancreatic ^35^ adenocarcinomas, SOX15 acts as a tumor suppressor and lack of SOX15 is associated with aberrant growth. The tumor suppressive action of SOX15 may be mediated through its downregulation of Wnt pathway components ^35, 44^. Notably, Wnt signaling promotes germline competence in mouse, pig, and human pluripotent cells, but after germline specification, excess Wnt signaling is detrimental ^8, 10, 45^. Since PRDM14 is also known to repress Wnt targets ^8^, the anticorrelation of *PRDM14* with respect to SOX15 perturbations may be a compensatory mechanism to maintain Wnt signaling within the range compatible with germline identity.

Overall, our research has identified that SOX15 is dispensable for establishing human germline identity, unlike SOX17, which is strictly required. Thus, our results do not support a role for SOX15 in the early stages of hPGC specification *in vivo*. However, we found that SOX15 promotes hPGCLC maintenance, and it may play a similar role in the human germline.

## Data availability

### Underlying data

Figshare: Supplementary Tables for “Testing the role of SOX15 in human primordial germ cell fate”. https://doi.org/10.6084/m9.figshare.9034232 ^27^.

This project contains the following underlying data:

- Extended Table 3 SOX15_SOX17_AID_raw (PGCLC fraction counts by flow cytometry).
- Extended Table 4 SOX15AID_timecourse_raw (PGCLC fraction counts by flow cytometry).
- Extended Table 5 SOX15DD_raw (PGCLC fraction counts by flow cytometry).
- Extended Table 6 qPCR_SOX15AID (ΔΔCt values for SOX15-AID).
- Extended Table 7 qPCR_SOX15DD_PGCLC (ΔΔCt values for SOX15 PGCLCs).
- Extended Table 8 qPCR_SOX15DD_SOMA (ΔΔCt values for SOX15 somatic cells).
- Extended Table 9 CT_raw (Raw Ct values).

Figshare: SOX15-Venus / OCT4 / BLIMP1 timecourse immunofluorescence raw images. https://doi.org/10.6084/m9.figshare.9104435 ^46^.

Figshare: myc-SOX15-DD / SOX17 immunofluorescence PGCLCs raw images. https://doi.org/10.6084/m9.figshare.9104471 ^47^.

Figshare: myc-SOX15-DD immunofluorescence ESCs raw images. https://doi.org/10.6084/m9.figshare.9104495 ^48^.

Figshare: SOX15-Venus / SOX17 / myc-TIR1 immunofluorescence SOX15-AID PGCLCs raw images. https://doi.org/10.6084/m9.figshare.9104504 ^49^.

Figshare: PRDM14-AID Venus / TIR1-DD ESCs immunofluorescence raw images. https://doi.org/10.6084/m9.figshare.9104666 ^50^.

Figshare: Raw FCS files for “Testing the role of SOX15 in human primordial germ cell fate”. https://doi.org/10.6084/m9.figshare.9385616 ^51^.

### Extended data

Figshare: Supplementary Tables for “Testing the role of SOX15 in human primordial germ cell fate”. https://doi.org/10.6084/m9.figshare.9034232 ^27^.

This project contains the following extended data:

- Extended Table 1 (oligonucleotides) (oligonucleotide primers used in this study).
- Extended Table 2 (primary antibodies for immunofluorescence).

Figshare: Extended Figure 1: Genotyping gels. https://doi.org/10.6084/m9.figshare.9248996 ^28^.

Figshare: Plasmids for “Testing the role of SOX15 in human primordial germ cell fate“. https://doi.org/10.6084/m9.figshare.9034199 ^26^.

Data are available under the terms of the Creative Commons Zero “No rights reserved” data waiver (CC0 1.0 Public domain dedication).
