# Supplementary material for: Testing the role of SOX15 in human primordial germ cell fate
Source: Wellcome Open Res. 2019 Sep 23;4:122. Originally published 2019 Aug 16. [Version 2] doi: 10.12688/wellcomeopenres.15381.2 (PMC6758833; doi:10.12688/wellcomeopenres.15381.2)
Supplement: Peer review history

# Peer review: Reviewer response for version 2

**DOI:** 10.21956/wellcomeopenres.16931.r36536

**Published:** 2019-09-23

**Authors:** Bernhard Payer

**Affiliations:** 1 Centre for Genomic Regulation (CRG), Barcelona Institute of Science and Technology (BIST), Barcelona, Spain; 2 Universitat Pompeu Fabra, Barcelona, Spain

The authors have addressed all points to my satisfaction.

I confirm that I have read this submission and believe that I have an appropriate level of expertise to confirm that it is of an acceptable scientific standard.

# Peer review: Reviewer response for version 1

**DOI:** 10.21956/wellcomeopenres.16803.r36242

**Published:** 2019-09-13

**Authors:** Elizabeth Robertson

**Affiliations:** 1 Sir William Dunn School of Pathology, Oxford University, Oxford, UK

This paper by Pieron Smela and colleagues addresses the potential role for the Sox family transcription factor Sox15 in specification of human primordial germ cells (PGCs).

 Interestingly both Sox 15 and the closely related family member Sox17 are highly expressed in human PGCs. Sox17 has previously been shown by this group to have an essential role on PGC specification, that seems to be unique for human PGCs, using *in vitro* protocols that induce the formation of so-termed human PGCLC from cultured hESCs using soluble cytokines. The current work tests the hypothesis that Sox15 may similarly be required for pluripotent cells to adopt this specialized cell fate. 

 Using a very well designed experimental strategy to efficiently remove Sox15 during *in vitro* specification of hPGCLC via an auxin-inducible degron system, the authors find that Sox15 depletion has no noticeable impact on the efficiency with which PGCLC can be induced at day 4. However, with time they show that the numbers progressively decrease. By contrast over-expression of Sox15 using a ProteoTuner approach had the reciprocal effect, namely an increase in the number of PGCLCs formed in the cultures. This observation was validated by q-PCR analysis of a set of selected genes. 

 As a control for the experimental system, and as a further proof of concept of the inducible/over-expression protocols presented, they nicely demonstrate that, as predicted, depletion of Sox17 completely abrogates PGCLC induction.

 Overall, the experiments summarized in the 5 Figures are very clearly displayed and the data and analysis are very clear. The conclusion that Sox15 is not essential for human PGC specification will be of interest to researchers in the field and the experimental protocols should be applicable for experiments designed to further tease apart the molecular circuitry governing the formation of human PGCLC *in vitro*. In my opinion the paper should be accepted as is without any revisions.

I confirm that I have read this submission and believe that I have an appropriate level of expertise to confirm that it is of an acceptable scientific standard.

# Peer review: Reviewer response for version 1

**DOI:** 10.21956/wellcomeopenres.16803.r36241

**Published:** 2019-09-02

**Authors:** Bernhard Payer

**Affiliations:** 1 Centre for Genomic Regulation (CRG), Barcelona Institute of Science and Technology (BIST), Barcelona, Spain; 2 Universitat Pompeu Fabra, Barcelona, Spain

In this paper, the authors investigated the role of SOX15, a transcription factor which is highly expressed in human primordial germ cells *in vivo* similar to its paralog SOX17, which is a known critical factor for human primordial germ cell-like cell (hPGCLC) differentiation from human embryonic stem cells (hESCs) *in vitro*. They thereby applied novel approaches like protein degradation by auxin-inducible degron (AID) technology and protein overexpression by the ProteoTuner system. Surprisingly, SOX15 seemed to be more important for maintenance of hPGCLCs instead of their specification. By testing the expression of genes in SOX15-depleted or overexpressing cells, they identified *PRDM14, SOX17* and *POU5F1* as potential differentially regulated genes. As these are factors with known roles in pluripotency and germ cell fate, these findings are potentially interesting and warrant further investigation.

 The study introduces new resources by generating novel cell lines allowing tuning the protein expression of the human germ cell factors SOX15 and SOX17. Thereby they will become useful tools for the community to address the functions of SOX15 and SOX17 in the human germ cell lineage.

**Specific comments**

1. Page 3, paragraph 2, last sentence: *“However, as opposed to mouse PGCs (mPGCs), the precise conditions required for hPGC maturation and epigenetic resetting remain unknown, and the current human PGCLC (hPGCLC) model system only allows study of cells in the pre-migratory state ^3,5^. " *
  It is not entirely true that in the human system only a pre-migratory state has been achieved. A recent study should be mentioned here (Yamashiro *et al*., (2018) ^1^), in which human PGCLCs, when co-cultured with mouse gonadal somatic cells, could advance to hPGCLC-derived oogonia displaying partial epigenetic reprogramming.
2. Page 3, paragraph 3: *“In mice, PGC fate is specified by a core network of three transcription factors: BLIMP1, PRDM14, and AP2γ ^2^.”*
  Here the paper by Nakaki *et al*., (2013) ^2^ should also be cited.
3. Figures 2C, D, 3C, 5B, 18: The Y-axis label indicating PGCLC induction efficiency fold change should be labeled better. In the figure legend, it needs to be stated, which antibody/reporter stainings were measured to define the PGCLC fold change in FACS. Additionally, it is not clear, why the control samples are not consistently set to 1 in these plots, but rather deviate from 1. The plots should be corrected, as by definition, the control should be set to 1 if the fold change in treated samples is to be compared to the control. 
4. Figure 2D: It seems that there is a much stronger effect on hPGCLC numbers on day 6, if SOX15 is depleted from day 0 onwards, when compared to depletion beginning at later time points, where the effect seems to not increase with longer time of SOX15 depletion (depletion from days 1-4 seem to have equal effect). This suggests there might be also a potential role for SOX15 during hPGCLC specification or right thereafter. The authors should consider this possibility. 
5. Figure 4D and page 8 paragraph 2: “ *Endogenous SOX15 expression was also downregulated on average, although this effect was not statistically significant (p = 0.08 after Holm- Bonferroni correction)*”. As the effect is extremely mild and not statistically significant, this sentence should be omitted.
6. Figure 4: The genes, which were significantly up- or downregulated after SOX15-depletion or overexpression changed mostly less than two-fold. Therefore these data need to be interpreted with caution, as the changes are only very mild. Unbiased genome-wide expression analysis by RNA-Seq could potentially identify more directly affected SOX15 targets and would provide more insight than focusing only on select candidates. The authors should tone down the interpretation of these expression data. 
7. Figure 5B+C. The figure legend is swapped between B+C. Please correct the mistake.

I confirm that I have read this submission and believe that I have an appropriate level of expertise to confirm that it is of an acceptable scientific standard.

## References

[1] : Generation of human oogonia from induced pluripotent stem cells in vitro. *Science*.2018;362(6412) : 10.1126/science.aat1674 356-360 10.1126/science.aat1674 30237246

[2] : Induction of mouse germ-cell fate by transcription factors in vitro. *Nature*.2013;501(7466) : 10.1038/nature12417 222-6 10.1038/nature12417 23913270

# Response

**Published:** 2019-09-16

**Authors:** Merrick Pierson Smela

We (the authors) have updated the article with a new version to address these comments:

*1) Page 3, paragraph 2, last sentence: “However, as opposed to mouse PGCs (mPGCs), the precise conditions required for hPGC maturation and epigenetic resetting remain unknown, and the current human PGCLC (hPGCLC) model system only allows study of cells in the pre-migratory state ^3,5^. "*

*It is not entirely true that in the human system only a pre-migratory state has been achieved. A recent study should be mentioned here (Yamashiro et al., (2018)), in which human PGCLCs, when co-cultured with mouse gonadal somatic cells, could advance to hPGCLC-derived oogonia displaying partial epigenetic reprogramming.*

We have included this reference, and clarified that the use of mouse gonadal somatic cells leaves unclear which precise factors are responsible for the maturation observed.

*2) Page 3, paragraph 3: “In mice, PGC fate is specified by a core network of three transcription factors: BLIMP1, PRDM14, and AP2γ ^2^.”*

*Here the paper by Nakaki et al., (2013) should also be cited.*

We have included this reference.

*3) Figures 2C, D, 3C, 5B, 18: The Y-axis label indicating PGCLC induction efficiency fold change should be labeled better. In the figure legend, it needs to be stated, which antibody/reporter stainings were measured to define the PGCLC fold change in FACS. Additionally, it is not clear, why the control samples are not consistently set to 1 in these plots, but rather deviate from 1. The plots should be corrected, as by definition, the control should be set to 1 if the fold change in treated samples is to be compared to the control.*

We have updated the Y-axis labels and figure legends to include more information.

The controls were not set to 1 since the comparisons for statistical tests were between the AID/TIR1 cell line fold change and the control cell line fold change, not between the AID/TIR1 cell line fold change and 1. This was explained in the Methods section under “Statistical Analysis.”

Ideally the control cell line fold change would be equal to 1, since in the absence of experimental variability and/or nonspecific effects the hPGCLC fraction would be the same with and without IAA. In our experiments the control cell line fold change did vary somewhat, but was not significantly different from 1 (one-sample Wilcoxon signed rank test, p > .05).

*4) Figure 2D: It seems that there is a much stronger effect on hPGCLC numbers on day 6, if SOX15 is depleted from day 0 onwards, when compared to depletion beginning at later time points, where the effect seems to not increase with longer time of SOX15 depletion (depletion from days 1-4 seem to have equal effect). This suggests there might be also a potential role for SOX15 during hPGCLC specification or right thereafter. The authors should consider this possibility.*

Although the decrease when SOX15 was depleted from day 0 seemed stronger, this was not significantly different from the decrease when SOX15 was depleted from days 1 – 4 (by Wilcoxon test, p = 0.11, 0.30, 0.30, 0.11). We have added a sentence to the paper which describes this.

Although this lack of significance does not by itself rule out a potential role for SOX15 during hPGCLC specification or right thereafter, in context of the data in Figure 2C we do not believe such a potential role is likely to be crucial. In any case, we do not believe this merits further discussion in the paper, especially given the next suggestion to remove a mention of a different statistically insignificant effect.

*5) Figure 4D and page 8 paragraph 2: “Endogenous SOX15 expression was also downregulated on average, although this effect was not statistically significant (p = 0.08 after Holm- Bonferroni correction)”. As the effect is extremely mild and not statistically significant, this sentence should be omitted.*

We have removed this sentence.

*6) Figure 4: The genes, which were significantly up- or downregulated after SOX15-depletion or overexpression changed mostly less than two-fold. Therefore these data need to be interpreted with caution, as the changes are only very mild. Unbiased genome-wide expression analysis by RNA-Seq could potentially identify more directly affected SOX15 targets and would provide more insight than focusing only on select candidates. The authors should tone down the interpretation of these expression data.*

We have added two sentences in the Discussion section mentioning these caveats. We agree that RNA-seq experiments would have provided more insight had we done them.

*7) Figure 5B+C. The figure legend is swapped between B+C. Please correct the mistake.*

We have corrected this.
